# Case report: Successful experience using continuous infusion of meropenem in a geriatric patient with hip fracture complicated by sepsis

**DOI:** 10.3389/fmed.2023.1148555

**Published:** 2023-04-27

**Authors:** Assiya Kadralinova, Assema Zh. Bekniyazova, Maiya E. Konkayeva, Aigerim A. Yeltayeva, Aidos K. Konkayev

**Affiliations:** ^1^Department of Anesthesiology and Intensive Care, Astana Medical University, Astana, Kazakhstan; ^2^Department of Anesthesiology and Intensive Care, National Scientific Center of Traumatology and Orthopedics named after Academician N.D. Batpenov, Astana, Kazakhstan

**Keywords:** hip fracture, sepsis, pneumonia, continuous infusion, meropenem, case report

## Abstract

This article highlights a clinical case of successful treatment of a 79-year-old multimorbid patient with a hip fracture resulting from a household injury. On the first day, the patient’s injury was complicated by infection and pneumonia. As a result, arterial hypotension, tachysystole, and respiratory failure progressed. With manifestations of sepsis, the patient was transferred to the intensive care unit. Surgical treatment in such a situation was contraindicated due to the high operational and anesthesiological risks, the unstable severe condition of the patient, as well as the presence of concomitant pathology in the form of coronary heart disease, obesity, and schizophrenia. According to the new sepsis management guideline, it was decided to use a continuous 24-h infusion of meropenem in addition to the complex treatment of sepsis. The use of continuous infusion of meropenem in this clinical situation may have caused the patient’s clinical improvement, which increased her quality of life and decreased the length of ICU stay and total hospital stay, despite an unfavorable cumulative prognosis and a high risk of in-hospital mortality.

## Introduction

Hip fracture is one of the most common pathologies in the elderly, which is often complicated by infection and the development of sepsis, which significantly worsens the prognosis of such patients. The incidence of hip fractures increases worldwide with the aging of the population and the development of osteoporotic changes, which creates problems for health systems due to concomitant diseases and a high risk of mortality (1).

Geriatric patients are more susceptible to infectious diseases and have a worse prognosis and higher mortality, partly due to a decrease in immune responses that occurs with age, known as immunosenescence (2, 3).

According to a systematic review published in 2016, more than 30 million cases of inpatient sepsis occur annually worldwide, with 5.3 million patients dying from sepsis. (4) Sepsis mortality continues to rise every year. In 2020, Rudd and colleagues reported 48.9 million cases of sepsis and 11.0 million sepsis-related deaths (5, 6). In another observational study, hospital mortality was 30% in patients with proven or suspected infection (7).

The time-dependent effect of meropenem and other beta-lactams has been studied for a long time. But in practice, only bolus (drug administration up to 30 min) or prolonged infusions (3–4 h) are used. Prolonged infusions are associated with a reduction in short-term mortality. Three meta-analyses reported similar results showing a reduction in short-term mortality (RR 0.70; 95% CI 0.57–0.87) with prolonged beta-lactam infusion (8–10).

According to the international guideline for the treatment of sepsis published in 2021, adults with sepsis or septic shock are encouraged to use a prolonged infusion of beta-lactams for maintenance therapy (after initial bolus administration) instead of bolus infusion (11). However, this guideline does not specify the optimal duration of infusion.

This clinical case deserves close attention as it highlights the effectiveness of the use of continuous infusion of meropenem in sepsis and the clinical improvement of the geriatric patient despite cumulative unfavorable prognosis in the absence of surgery for a hip fracture. According to the CFS (Clinical Frailty Scale), the patient scored 8 points, followed by the SIRS 2 points, the qSOFA 2 points, and the SOFA scale 5 points, which is associated with a high risk of in-hospital mortality, as well as a long stay in the ICU and in the hospital.

## Case description

### Patient information

A 79-year-old female patient was urgently admitted to the hospital with a hip fracture resulting from a household injury. The injury happened 6 h ago. At the time of admission, the patient experienced pain, impaired movement, and support inability in the right lower limb. From the past medical history, it is known that the patient has been registered for arterial hypertension with a cardiologist and for schizophrenia with a psychiatrist for more than 50 years, and regularly takes haloperidol and situationally antihypertensives. According to the patient’s daughter, the last hospitalization for mental illness was in 1979. Heredity and allergic anamnesis without features. She underwent surgery for a total hysterectomy more than 15 years ago, and for an inguinal herniotomy 10 years ago. Menopause since the age of 50 years. The patient was taken to the hospital by an ambulance and received prehospital care for the immobilization of the right lower limb and analgesia.

### Clinical findings

According to the initial check-up, the patient suffered from class 1 obesity with a weight of 98 kg (BMI = 33.9 kg/m^2^). The visible skin areas were pale pink in color. The patient’s initial body temperature was 36.4\u00B0C. Breathing of the patient was spontaneous (17–18 per min) with vesicular breathing in lungs auscultated in all fields, and SpO2 was 91%. The patient’s heart tones were muffled and rhythmic. Blood pressure reached 110/96 mmHg with a pulse of 73 beats per minute. Furthermore, the patient’s abdomen was not swollen and soft on palpation; urination was independent and free at the time of the checkup. Locally, visual deformation of the affected area was not observed in the patient. Palpation of the right hip joint was presented by sharp pain and the axial load was painful. After 11 h of admission time, the patient’s state sharply deteriorated.

### Timeline

**Figure 1:**
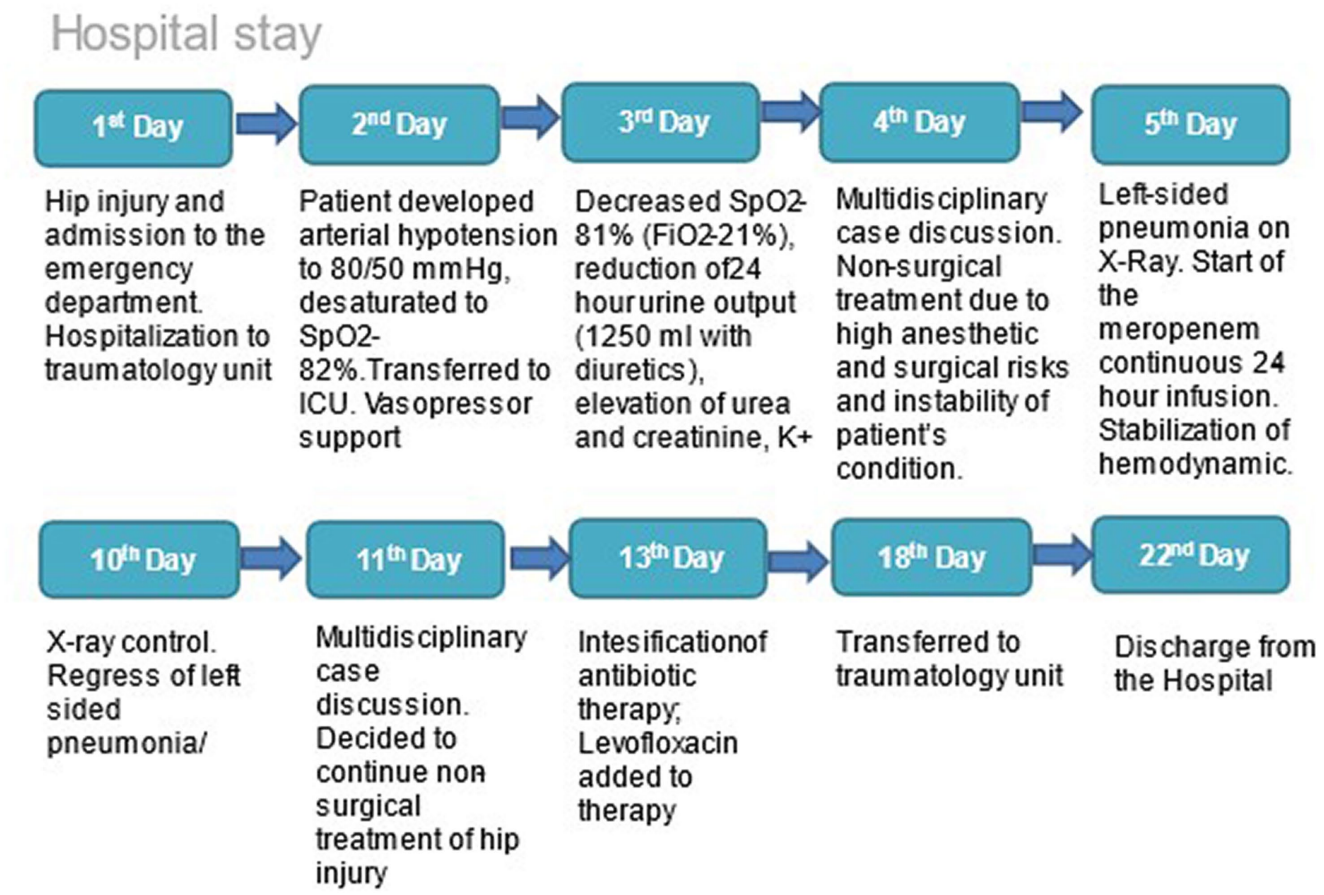
Chronology of the patient’s medical history from the moment of admission to discharge from the hospital.

The chronology of the patient’s medical history from the time of admission to the time of discharge from the hospital is highlighted in Figure 1.

### Diagnostic assessment

**Figure 2:**
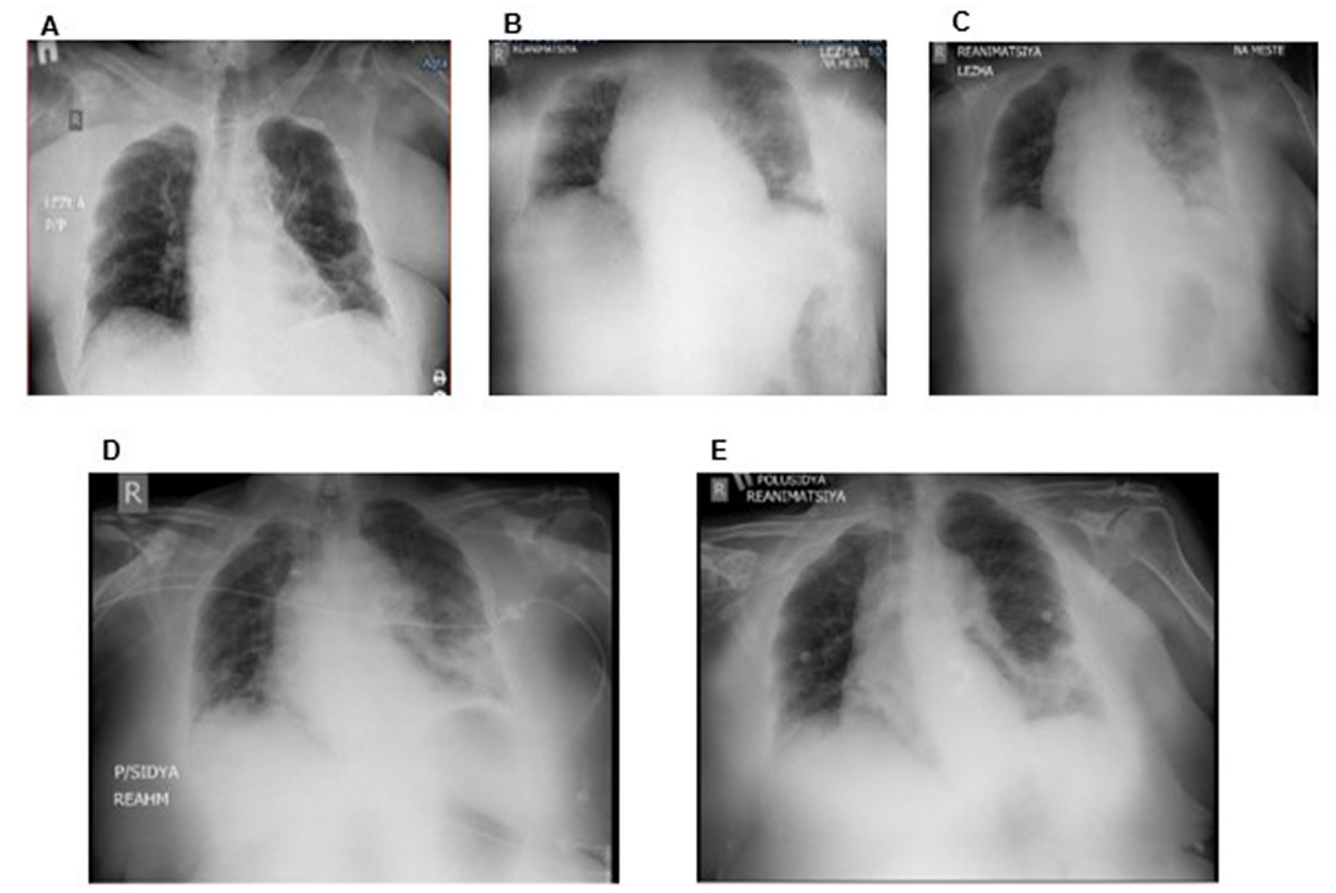
Dynamics of chest X-ray after adding a continuous infusion of meropenem: CXR on the 1st day **(A)**, CXR on the 2nd day **(B)**, CXR on the 3rd day **(C)**, CXR on the 5th day **(D)**, and CXR after 6 days of continuous infusion of meropenem (11th day of hospitalization) **(E)**.

As a result of initial routine laboratory tests of the patient, the following changes in the form of leukocytosis with a stab shift of the leukoformula to the left, accelerated ESR, and mild anemia were revealed in CBC. The urine test of the patient also showed inflammatory changes. Blood biochemistry revealed an impaired renal function in the form of increased levels of creatinine and urea, slightly increased hepatic transaminases, and hypoproteinemia. The hemostatic system showed no gross violations throughout the entire period, sometimes coagulogical results were with a slight hypocoagulation trend. On admission, a chest X-ray showed cardiomegaly and no visible changes in the lungs (Figure 2A). After 11 h of admission, respiratory was progressed in the patient, and the control X-ray of the chest showed left-sided pneumonia (Figure 2B). The patient was urgently transferred to the ICU, and blood gas analysis showed a low oxygenation index (270.3 mm Hg). PCR test for COVID-19 was negative. In dynamics, on the 3rd day of hospital stay, according to the results of the chest X-ray, pneumonia progressed that required intensification of antibiotic therapy (Figure 2C). Continuous infusion of meropenem (Santo, Kazakhstan, Shymkent) was added to the complex therapy of sepsis. On the 5th day of hospital stay, a chest X-ray had shown positive changes in the condition mentioned above (Figure 2D). On the 11th day of hospital stay, after receiving continuous infusion of meropenem for 6 days as part of complex treatment, the chest radiograph had shown resolution of left-sided pneumonia (Figure 2E).

The result of the initial ECG was relatively normal with sinus rhythm and a heart rate of 89 beats per minute and a normal electrical axis of the heart. Moreover, cardiac ultrasound showed moderate hypertrophy of the LV walls and LV diastolic dysfunction with global myocardial contractility of 60%. Ultrasound dopplerography of lower limbs and brachiocephalic trunk, ultrasonography of the abdominal organs, and pelvic ultrasound showed no changes. Only an ultrasound of the pleura and pleural cavities showed effusion on both sides in a volume of 100–150 mL. However, it was decided to refrain from puncture of the pleural cavity (100–150 mL on both sides) due to the absence of an increase in pleural effusion in dynamics and a high risk of complications.

The excretory function of the kidneys was also impaired and manifested as dysuric disorder and increased levels of urea, creatinine, and potassium in biochemistry. Against the background of complex therapy using continuous infusion of meropenem and diuretics, kidney function was significantly improved.

The cultures were obtained before the appointment of meropenem, on the 3rd day and the 8th day after meropenem prescription, and before discharge. A respiratory sample and blood and urine samples were cultured. According to the results of the first crops, no growth was found in any of the samples. Based on culture results on day 3 after meropenem administration, Candida Albicans 10^5 was detected in the urine, and antifungal therapy was prescribed according to sensitivity. Further crops also showed no growth of fungus and other microorganisms in any of the samples. Fluoroquinolones were prescribed as an enhancement of antibiotic therapy taking into account persistent leukocytosis, which appeared as leukocyturia (suspected infection of the lower urinary tract).

Also, the patient was diagnosed with post-traumatic anemia, which progressed on the 10th day of hospitalization and required sessions of blood transfusion therapy for several days. Sepsis was detected as a result of the patient’s hip injury followed by infection and dysfunction of the lungs and kidneys, as well as hemodynamic disorders.

Based on the foregoing, the patient was diagnosed with a closed pertrochanteric fracture of the right femur with a displacement of bone fragments. Moreover, such complications as sepsis, left-sided pneumonia, complicated by respiratory failure and pleural effusion, severe anemia, and impaired renal function were found in the patient.

In addition to the above, the patient has comorbidities: paranoid schizophrenia; state of medical remission; ischemic heart disease; 2-degree arterial hypertension, risk 3; chronic heart disease, functional class 1; chronic kidney disease; impaired glucose tolerance; class 1 obesity (BMI = 33.9 kg/m^2^); postoperative reducible ventral hernia of the mesogastric region; and transient disturbance of the passage through the intestines.

### Therapeutic intervention

Based on the results of the two multidisciplinary case discussions, it was decided to refrain from performing surgery due to high anesthetic and surgical risks, as well as the unstable general condition of the patient and the presence of severe comorbidities (obesity, coronary artery disease, and chronic heart failure). The hip fracture was treated conservatively using a derotational boot; subsequently, an immobilization plaster bandage was applied to the right lower limb.

### Oxygen therapy

After 11 h of admission, the patient developed acute respiratory failure requiring an unplanned transfer to the intensive care unit. Oxygen insufflation *via* nasal cannulas was urgently connected at a rate of 6 L/min. A gas blood test revealed a decrease in the oxygenation index (P/*F* = 270.3 mmHg). During almost all days in ICU, oxygen therapy was an integral part of complex therapy. After adding continuous infusion of meropenem to the complex therapy, the patient’s respiratory function significantly improved and oxygen demand decreased. Moreover, 2 days before the transfer of the patient from the intensive care unit to the specialized department, the oxygenation index was relatively normal in the absence of oxygen support (P/*F* = 314.9 mmHg).

### Anti-infection therapy

On 1st day, the patient was empirically prescribed to take cefazolin 1 g intravenously thrice daily, taking into account the initial laboratory tests (neutrophilic leukocytosis, shift to the left of leukoformula). Subsequently, on the 5th day, due to the deterioration of the patient’s condition, the development of sepsis, and the ineffectiveness of cephalosporine therapy, it was necessary to change antibiotic therapy to meropenem (Santo, Kazakhstan, Shymkent). Meropenem is a carbapenem with a broad spectrum of activity against a great variety of gram-positive and gram-negative pathogens. Its good penetration into fluids and tissues makes meropenem a good choice for the treatment of severe infections. The patient was prescribed continuous 24-h infusion therapy with 3 g/day of meropenem through a perfusor after 1 g of meropenem bolus. A dose of 3 g/day was prescribed in accordance with the relatively preserved renal function. Since the cultures showed no growth in any of the specimens, MICs could not be determined and we were guided by the results of clinical data and laboratory findings, picture in the lungs. The continuous infusion was provided as follows: 500 mg of meropenem was diluted in 50 mL (10 mg/mL) of NaCl 0.9% solution at 12.5 mL/h. After a week of treatment with meropenem, antibacterial therapy was intensified—levofloxacin 500 mg twice daily intravenously was added. Fluoroquinolones were prescribed as an enhancement of antibiotic therapy taking into account persistent leukocytosis, which appeared as leukocyturia (suspected infection of the lower urinary tract).

Additionally, the patient took fluconazole 100 mg per day enterally.

### Glucocorticoid therapy

The patient also received dexamethasone at a dosage of 4 mg twice daily for 3 days.

### Anticoagulant therapy

To prevent thromboembolic complications, the patient received nadroparin sodium 30 mg subcutaneously once daily.

### Pain management

Analgin 100 mg, ketoprofen 100 mg, or promedol 20 mg was used situationally for pain management.

### Liquid volume management

Transfusion therapy with blood components was performed on the patient as well using washed erythrocytes to correct anemia and freshly frozen plasma, and 20% albumin solution to correct hypoproteinemia.

The volemic status and hydrobalance were assessed daily, and if necessary, diuresis was stimulated with furosemide intravenously at a dosage of 20 mg twice a day.

### Nutritional support

The patient also received nutritional support at the rate of 25–30 kcal/kg/day, and protein provision of 1.2–1.5 g/kg/day.

### Other

The patient received basic antipsychotic therapy: haloperidol 5 mg once daily and cyclodol 2 mg once daily after consultation with a psychiatrist. Correction of water-electrolyte disorders, gastroprotective therapy, symptomatic therapy—antiemetic drugs (ondansetron), improvement of the passage of contents through the intestine (lactulose), intestinal atony (proserin), sedative therapy, broncholytic therapy (ambro and eufillin), and correction of hyperglycemia (insulin) were also carried out as necessary. The patient also received vasopressor support of hemodynamics with dopamine for 4 days, and antiarrhythmic therapy (bisoprolol 5 mg) under heart rate control. Furthermore, vascular therapy also was carried out in patients to improve blood microcirculation. Additionally, the patient received sessions of vibroacoustic lung therapy (VALT) according to the scheme, respiratory gymnastics, and early rehabilitation therapy.

### Follow-up and outcomes

**Figure 3:**
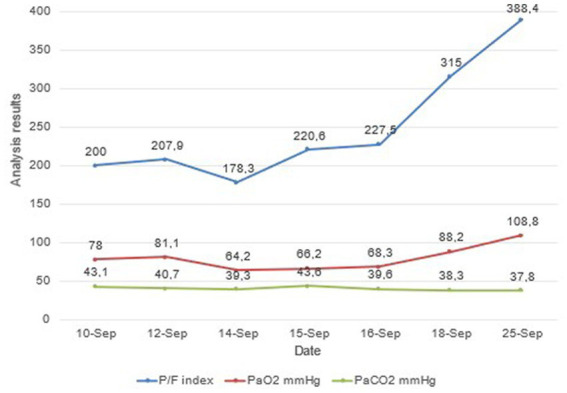
The dynamics of the patient’s respiratory function indicators.

After receiving the informed consent of the patient and the consent of her daughter, the tactics of continuous infusion therapy with meropenem in the amount of 3 g/day through a perfuser after bolus administration of 1 g of meropenem was applied. After starting a 24-h infusion of meropenem, the patient showed a gradual improvement in respiratory function, as seen in Figure 3.

**Figure 4:**
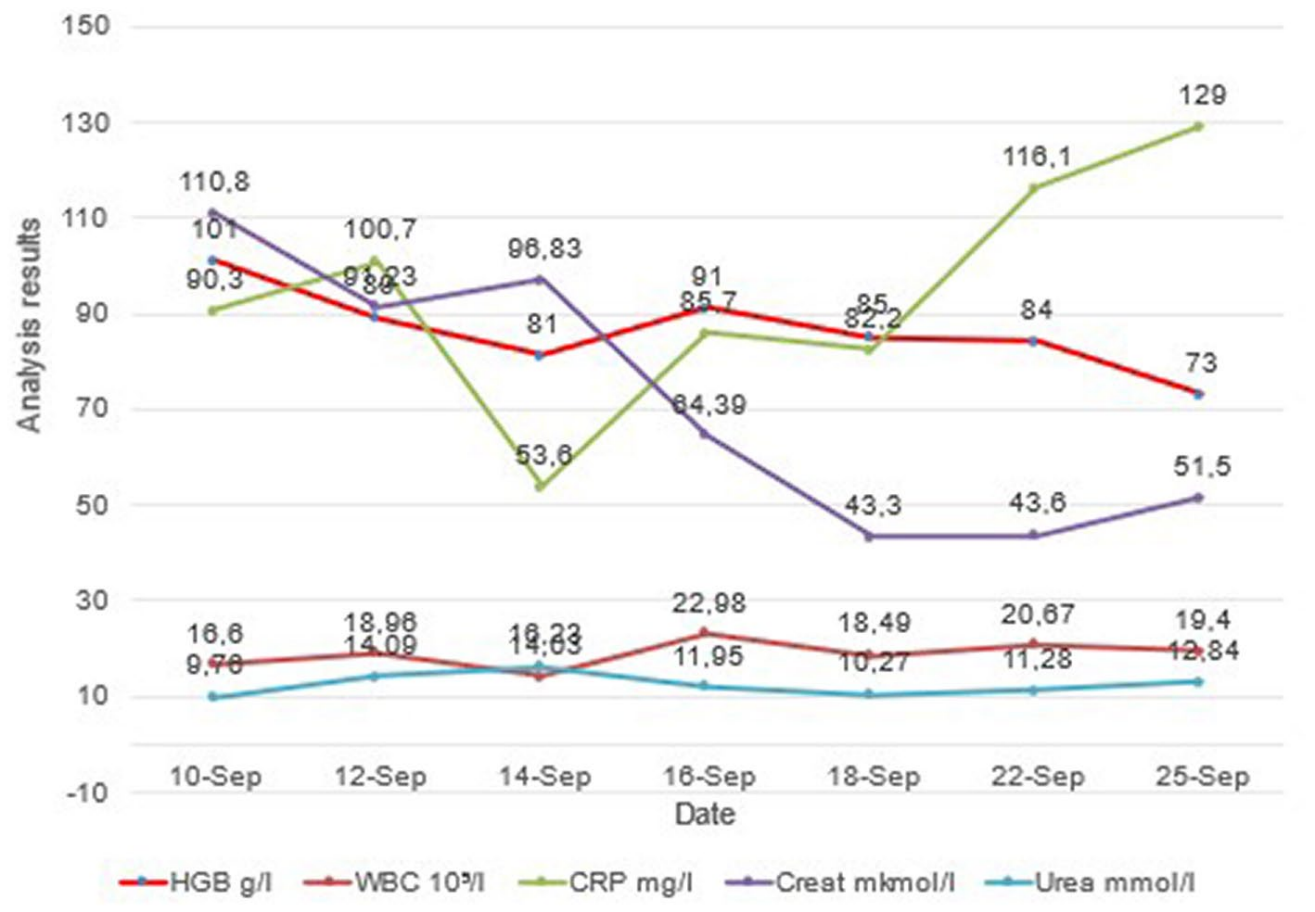
The dynamics of tests from the moment of the patient’s admission to the discharge.

However, indicators of CRP and leukocytes were relatively stable and did not decrease in dynamics, despite significant improvement in respiratory function; the clinical picture of the patient is shown in Figure 4. After stabilizing the patient, improving respiratory function, and stabilizing hemodynamics and excretory kidney function, it was decided to transfer the patient to a specialized department.

The patient was transferred to a specialized department in a stable condition, not requiring oxygen support, and was discharged from the hospital after 5 days for rehabilitation treatment in the outpatient clinic.

## Discussion

It is expected that geriatric trauma patients will have more comorbidities and correspondingly higher rates of mortality and morbidity. Moreover, patients with geriatric trauma who require unscheduled admission to the intensive care unit may have worse outcomes (12, 13).

Our 79-year-old patient had the most unfavorable prognosis: according to the CFS (Clinical Frailty Scale), used to assess senile asthenia, the patient scored 8 points, followed by the SIRS 2 points, the qSOFA 2 points, and the SOFA scale 5 points, which is associated with a high risk of in-hospital mortality and prolonged stay in the ICU and in the hospital.

According to Andrew Li and Jason Phua’s study of sepsis in Asia, sepsis is a common cause of hospitalization in Asian intensive care units. Mortality remains high and higher in low- and middle-income countries after confounding factors are excluded. The overall prevalence of sepsis in intensive care units was 22.4% and the hospital mortality rate was 32.6% (14).

This clinical case is indicative because, after the start of a continuous 24-h infusion of meropenem in the complex treatment of sepsis, there was significant clinical improvement in the parameters of a geriatric multimorbid patient with sepsis. Such a situation allowed to transfer the patient for further management in the conditions of the specialized department after 12 days and further, after another 5 days to discharge home to continue rehabilitation and treatment in the outpatient setting.

Because of the patient’s unstable condition in the first 48 h, the operation could not be performed, as recommended in the observational study of Leer-Salvesen S et al. According to the conclusions of the authors, mortality remains unchanged when the total delay of the operation is less than 48 h. A total delay of more than 48 h is associated with an increased risk of 3-day mortality (15, 16). But even after the patient’s condition stabilized, the risk of perioperative complications exceeded the expected benefit from the operation, and it was decided to manage the patient conservatively.

Complications in the form of infections often accompany such clinical cases and multiply the mortality rate, increasing the length of stay in the ICU, which increases the economic costs and reduces the patient’s quality of life due to their stay in the intensive care unit.

According to our search of the scientific literature, the pharmacokinetic and pharmacodynamic features of the use of continuous infusions of carbapenems are very well studied in pediatric practice (17, 18) and have long been implemented in pediatric practice. Since the study in the pediatric population is carried out separately in view of the presence of metabolic features of the child’s body, geriatric patients also deserve special attention and should also be considered separately from the younger population due to the presence of age-related features. Studies in gerontological patients are few and the main issue is the optimal dosing of drugs due to reduced kidney function in the elderly.

Muhammad Usman et al. in their study concluded that a continuous infusion of 3,000 mg daily dose is preferred if clearance of creatinine Cl_Cr_ > 50 mL/min in elderly patients (in this case, patient’s Cl_Cr_ = 54 mL/min) (19).

O’Jeanson A et al. in their study concluded that the continuous infusion mode (with loading dose) allowed the obtaining of the pharmacokinetic/pharmacodynamic target for a larger number of patients (100% for MIC ≤20 mg/L). For the treatment of susceptible bacteria (MIC ≤2 mg/L), differences in the probability of target attainment among bolus-like, extended, and continuous infusions were negligible. (20) Additionally, in the other prospective study, the authors concluded that prolonged infusion time was not always beneficial for those who needed a higher therapeutic target (100% fT > MIC, 100% fT > 4 MIC) or with MIC > 4 mg/L. (21). In the systematic review of the pharmacokinetics of β-lactams, authors concluded that continuous infusion does not improve outcomes (22).

This clinical case reflects the use of continuous meropenem infusion in a gerontological severe patient with hip injury and sepsis. We observe clinical improvement in the patient, but the laboratory data of infection and inflammation do not change much (leukocytosis, CRP). Persistent leukocytosis and an increase in CRP levels, in this case, are probably associated with decreased immunoreactivity against the background of an existing hip injury, age, and comorbidity (obesity, hyperglycemia, and hypertension). An increase in the P/F index and SpO2 and a decrease in oxygen dependence, together with regression of dyspnea and an improvement in the X-ray picture of the lungs, allowed us to conclude that there was a clinical improvement with the use of a 24-h meropenem infusion. Therefore, there is a need for further clinical studies on the continuous use of carbapenems in gerontological patients.

## Patient perspective

Due to delirium and manifestations of encephalopathy of mixed genesis, it was difficult to obtain the patient’s opinion on the treatment being taken. But based on observations of the patient’s condition on the 2nd day after the start of continuous infusion of meropenem therapy as part of complex treatment, the patient began to breathe easier.

## Author’s note

The authors have read the CARE Checklist (2013), and the manuscript was prepared and revised according to the CARE Checklist (2013).

## Data availability statement

The original contributions presented in the study are included in the article/Supplementary material, further inquiries can be directed to the corresponding author.

## Ethics statement

Ethical review and approval was not required for the study on human participants in accordance with the local legislation and institutional requirements. The patients/participants provided their written informed consent to participate in this study. Written informed consent was obtained from the individual for the publication of any potentially identifiable images or data included in this article.

## Author contributions

AKo, MK, and AY: conceptualization and organization of the database. AB: writing draft. AKa: review and editing of the manuscript. All authors contributed to the article and approved the submitted version.

## Conflict of interest

The authors declare that the research was conducted in the absence of any commercial or financial relationships that could be construed as a potential conflict of interest.

## Publisher’s note

All claims expressed in this article are solely those of the authors and do not necessarily represent those of their affiliated organizations, or those of the publisher, the editors and the reviewers. Any product that may be evaluated in this article, or claim that may be made by its manufacturer, is not guaranteed or endorsed by the publisher.
